# Auditory function in humans at high altitude. A scoping review

**DOI:** 10.1371/journal.pone.0291919

**Published:** 2023-09-21

**Authors:** Michela Masè, Andrea Viziano, Giacomo Strapazzon, Marco Alessandrini, Alessandro Micarelli

**Affiliations:** 1 Institute of Mountain Emergency Medicine, Eurac Research, Bolzano, Italy; 2 Laboratory of Biophysics and Translational Cardiology, Department of Cellular, Computational and Integrative Biology–CIBIO, University of Trento, Trento, Italy; 3 Department of Clinical Sciences and Translational Medicine, ENT Unit, University of Rome Tor Vergata, Rome, Italy; 4 Unit of Neuroscience, Rehabilitation and Sensory Organs, UNITER ONLUS, Rome, Italy; University of Southern Mississippi, UNITED STATES

## Abstract

**Purpose:**

High-altitude (HA) affects sensory organ response, but its effects on the inner ear are not fully understood. The present scoping review aimed to collect the available evidence about HA effects on the inner ear with focus on auditory function.

**Methods:**

The scoping review was conducted following the guidelines of the Preferred Reporting Items for Systematic Review and Meta-Analysis extension for scoping reviews. PubMed, Scopus, and Web of Science electronic databases were systematically searched to identify studies conducted in the last 20 years, which quantified in healthy subjects the effects of HA on auditory function.

**Results:**

The systematic search identified 17 studies on a total population of 888 subjects (88.7% male, age: 27.8 ± 4.1 years; median sample size of 15 subjects). Nine studies were conducted in a simulated environment and eight during real expeditions at HA. To quantify auditory function, six studies performed pure tone audiometry, four studies measured otoacoustic emissions (OAE) and eight studies measured auditory evoked responses (AER). Study protocols presented heterogeneity in the spatio-temporal patterns of HA exposure, with highly varying maximal altitudes and exposure durations.

**Conclusion:**

Most studies reported a reduction of auditory function with HA in terms of either elevation of auditory thresholds, lengthening of AER latencies, reduction of distortion-product and transient-evoked OAEs. Future studies in larger populations, using standardized protocols and multi-technique auditory function evaluation, are needed to further characterize the spatio-temporal pattern of HA effects along the auditory pathways and clarify the pathophysiological implications and reversibility of the observed changes.

## 1. Introduction

High-altitude (HA), defined as an altitude higher than 2500 m above sea level, is mainly characterized by conditions of reduced oxygen (hypoxia) and atmospheric pressure (hypobaria) with respect to sea level, where reference levels of oxygen (normoxia) and atmospheric pressure (normoxia) are present [1]. These conditions elicit multiple physiological responses and adaptation mechanisms, but they may also result in maladaptive processes causing illness (e.g., acute mountain sickness (AMS)). The wide spectrum of responses depends on several factors, which include the duration and the altitude of exposure, as well as the rate of ascent and acclimatization pattern [2]. The effects of acute hypoxia, mainly mediated through the stimulation of chemoreceptors, involve a series of cardiovascular, respiratory, and cerebrovascular responses, which are aimed at mitigating the decrease in oxygen with HA [1]. Although still debated, current evidence suggests also a role of hypobaria in physiological alterations. Hypobaric hypoxia (HH) seems to produce more severe effects than normobaric hypoxia (NH) at a given inspired oxygen pressure, determining lower oxygen saturation levels and amplified cascade effects [3, 4]. HA-induced alterations may impact cerebral blood flow, which regulates oxygen delivery to the brain and is sensitive to hypoxia and hypocapnia through vasoactive responses of the middle cerebral artery [5, 6]. The brain is particularly dependent on oxygen and thus it may be exposed to severe consequences in hypoxia, when compensatory mechanisms are insufficient [1, 7]. Neurological effects may span from headache and dizziness to impairment of perceptual and cognitive processes when higher cortical regions are involved [8]. Focusing on sensory organs, vision is the first sense to be altered by the lack of oxygen and has been mainly investigated [9]. Conversely, the effects of HH on the ear and, specifically, on auditory function are less clear [10], due to a relatively small number of studies, often with conflicting results [2, 10–16]. Discrepancy in results may arise from different factors, related to differences in experimental protocols and altitude spatio-temporal profiles, in the methods used for auditory testing, and in the interaction between these two aspects. Different techniques may have diverse sensitivity to the stages and pathways of auditory processing. On the other hand, different regions involved in audition may be diversely susceptible to the effect of hypoxia in terms of magnitude and mechanisms [11, 15, 16]. Environmental conditions, such as lower air pressure (i.e., hypobaria), may exert physical effects and alter the mechanisms of sound transmission and transduction, affecting measurements such as pure tone auditory thresholds [10]. Together with experimental variability factors, data interpretation is further complicated by the incomplete knowledge of the mechanisms of oxygen supply to the inner ear and the effects of hypoxia on these mechanisms [11, 17, 18]. Finally, a reduction in cognitive performance may by itself have repercussions on auditory function, when this is investigated via subjective tests [19].

The aim of this scoping review was to collect the literature from the last 20 years about HA effects on the inner ear and auditory function. Given the above premises, specific attention was devoted to the description of HA exposure protocols, including both simulated and in-field HA conditions, as well as of the techniques used to evaluate auditory function. The review is structured as follows: a brief overview on the techniques available to investigate general auditory function or specific auditory pathways is provided in Section 1. Section 2 defines the methods applied to conduct the scoping review. The results of the search and the selected studies are described in Section 3 and critically discussed in Section 4.

### 1.1 Techniques for auditory function testing

**Fig 1:**
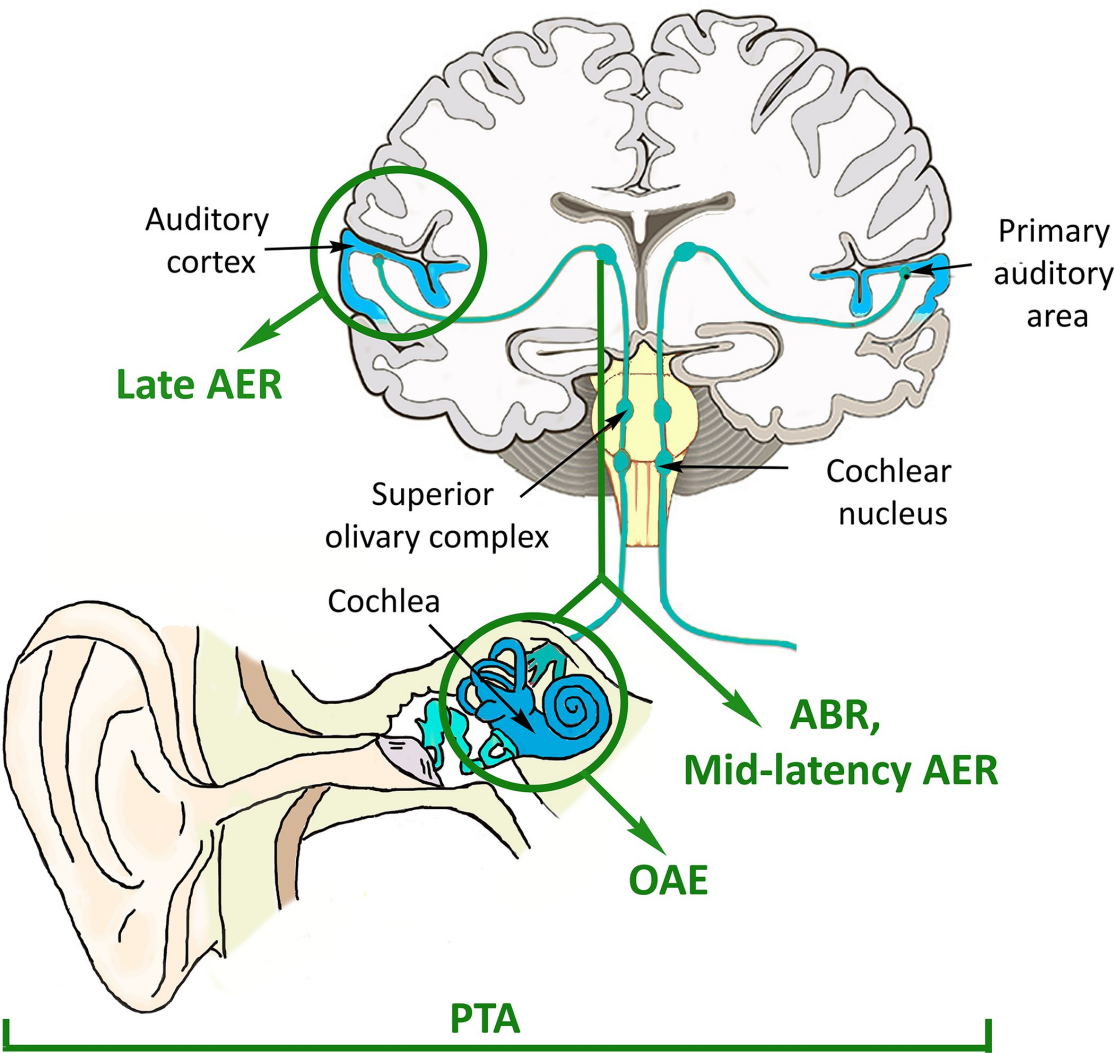
Techniques for the assessment of auditory functions at different levels of the auditory pathways. Anatomic scheme of the ear and auditory pathways to the brain. Green arrows and circles indicate the areas of the auditory pathways, which are assessed by different auditory techniques. ABR, auditory brainstem response; AER, auditory evoked response; OAE, oto-acoustic emissions; PTA, pure tone audiometry.

Different techniques are available for audiological testing. These provide an overall evaluation of auditory function or a focused appraisal of specific areas along the pathway from the cochlear region to higher brain areas (Fig 1). In the context of the present review, pure tone audiometry (PTA) measures hearing thresholds (i.e., the lowest intensity at which a pure tone can be heard across a range of human audible frequencies) and, in the case abnormalities are detected, distinguishes if they arise from conductive or sensorineural hearing loss. PTA provides a subjective measure of hearing performance, by evaluating the entire auditory pathway from the external ear to central processing [20].

Otoacoustic emissions (OAE) and auditory evoked responses (AER) tests provide objective and more specific measures of hearing [20]. OAE are sounds generated from the mechanical components of the cochlear hair cells. These signals are transmitted across the middle ear to the external ear canal, where they can be recorded [21]. The production of OAE is a marker of inner ear health and a simple way to screen for hearing loss [22]. There are two types of OAE: spontaneous OAE, which occur without external stimulation, and evoked OAE, which require an acoustic stimulus to be delivered. Transient-evoked OAE (TEOAE) are evoked using click or tone-burst stimuli and provide an overall view of cochlear function across a broad range of frequencies [20]. Click stimuli cover a large frequency range, up to 4 kHz, to evoke responses from multiple nerve fibers [23]. Tone-burst stimuli are delivered in a narrower frequency range, mostly at lower frequencies, to obtain frequency-specific responses [24]. Distortion-product OAE (DPOAE) are evoked using two simultaneous pure tone stimuli (referred as f1 and f2). Unlike TEOAE, DPOAE are customized to assess frequencies that match the patient’s audiogram and are more sensitive to detect high-frequency hearing loss [25], since the combination of selected tones activates a specific cochlear portion [26].

AER evaluate electrical activity related to auditory neurons, both at lower auditory level (auditory brainstem responses [ABR] audiometry) and at the central level (middle-latency responses, late AER, auditory cognitive or steady-state responses [SSR]), through the registration of electroencephalographic (EEG) signals under specific protocols and paradigms [27]. ABR record the electrical activity from the eighth cranial nerve to neurons along the brainstem auditory pathway in response to an acoustic stimulus. Tracings are composed of a sequence of up to seven positive wave peaks (labeled I-VII) interspersed with negative troughs, which occur 0–8 ms after stimulus onset. There is still debate about the regions generating different ABR waveforms, except for wave I that is generally attributed to the cochlea. Wave II is thought to arise from the cochlear nucleus, whilst waves III, IV, and V from generator sites within the brainstem and the midbrain. Middle-latency AER are successive waves of negative and positive voltage (labeled with the letter N and P, respectively), which occur 8–50 ms after stimulus onset. These responses are usually analyzed in the study of central auditory disorders and are presumably generated within thalamocortical pathways to the auditory cortex [20]. While ABR and middle-latency AER indicate the trajectory of sound from the cochlea to the brainstem and subcortical areas, long latency AER or late AER reflect perceptual and cognitive processes from higher brain regions in response to auditory events [28]. Late AER occur in a time window >50 ms after stimulus onset and are sub-classified into earlier or exogenous (e.g., P50, P100, N100, P200, and N200), and later or endogenous (e.g., P300) [20]. Exogenous late AER are necessarily triggered by an external stimulus and are mainly influenced by the physical characteristics of the stimulus, such as intensity, frequency, and duration [28]. Endogenous late AER mainly reflect task-dependent neural processes. For instance, the P300 is a large parieto-central positive late AER, which occurs when an expected, but relevant and informative stimulus is detected and the neuronal model of the environment needs to be consistently updated [29]. Finally, auditory SSR, are elicited by repetitive auditory stimuli (usually at 40 Hz) and originate in the primary auditory cortex [30].

## 2. Methods

The scoping review was conducted following the guidelines of the Preferred Reporting Items for Systematic Review and Meta-Analysis (PRISMA) extension for scoping reviews (PRISMA-ScR) [31].

### 2.1 Eligibility criteria

The literature search was performed by two authors (AM and MM) to identify studies, conducted in the last 20 years, that analyzed the effects of HA exposure on the human inner ear, with specific focus on the auditory function. The search strategy is schematized by the inclusion criteria in Table 1, categorized according to the recommended Population-Concept-Context (PCC) mnemonic for scoping review [32, 33].

**Table 1:** Inclusion criteria for the scoping review summarized according to the Population-Concept-Context (PCC) mnemonic, recommended for scoping reviews [32, 33].

**Population**	• Awake healthy adults (no ear disease, no respiratory disease). • Any gender.
**Concept**	• Effects of high altitude on inner ear physiology. • Quantitative evaluation of auditory function.
**Context**	• Exposure to simulated (hypoxic, hypobaric, or combined conditions) or real high-altitude conditions, in either acute or chronic exposure settings. • Original peer-reviewed research articles (any study design), published in English in the last twenty years.

The scoping review addressed HA exposure and inner ear function in awake healthy adults. Studies in children or in patients with pre-existing ear disease or ear implants, and studies performed in conditions of hypoxia due to the presence of respiratory diseases (e.g., sleep apnea, chronic obstructive pulmonary disease, etc) were excluded. We included studies that quantified auditory function in the inner ear and/or at the central level, applying techniques such as PTA, AER, and OAE. Studies focusing solely on the middle ear, on vestibular function, or higher cognitive responses were excluded. In terms of HA environmental conditions and exposure duration, we included studies in either simulated (including exposure to hypoxia, hypobaria, or both conditions) or real HA environments (including both acute and chronic exposure duration). Studies analyzing the effects of atmospheric pressure changes on the inner ear in simulated or real flights were included, while pressure changes associated with diving and hyperbaric contexts, or with sudden pressure changes in parabolic flights, centrifuge simulations, space flights, hyper-gravity, or microgravity were excluded. The search was restricted to articles published in English in peer-reviewed journals. No restriction on study design was posed. Abstracts presentations, conference proceedings, and reviews were excluded.

### 2.2 Information sources, search strategy, and study selection

A systematic search was performed in Pubmed, Scopus, and ISI Web of Science electronic databases to identify primary references from January 2002 to June 2022. The following search strings were used: ("otoneurology" OR "neurotology" OR "otorhinolaryngology" OR "ear" OR "inner ear" OR "tinnitus" OR "cochlea" OR "cochlear" OR "auditory" OR "acoustic" OR "ENT" OR "deafness" OR "hearing") AND ("altitude" OR "high altitude" OR "mountain" OR "hypoxia" OR "hypoxic" OR "hypobaria" OR "hypobaric" OR "atmospheric pressure" OR "low atmospheric pressure" OR "simulated altitude" OR "flight" OR “simulated flight”) NOT ("dive" OR "diving" OR "divers" OR "hyperbaria" OR "hyperbaric" OR “microgravity” OR “hypergravity” OR “space flight” OR “parabolic flight”). The database search was followed by a review of the citations from eligible studies. Studies were selected based on title and abstract using the online platform Rayyan [34]. Selected studies were read thoroughly to identify those suitable for inclusion in the scoping review.

### 2.3 Data extraction

Two reviewers (AM and MM) independently extracted the demographic and experimental data from the selected studies. When disagreement occurred, they reviewed the papers together to reach consensus. For each study, the following relevant information was extracted and summarized: the characteristics of the investigated study population, the HA protocol (altitude level, exposure duration, and pattern of the exposure) and environmental condition (simulated or real HA, presence of hypoxia and/or hypobaria), the technique used to assess inner ear function, and the main results of the studies with focus on HA effects on the auditory function.

## 3. Results

### 3.1 Study selection

**Fig 2:**
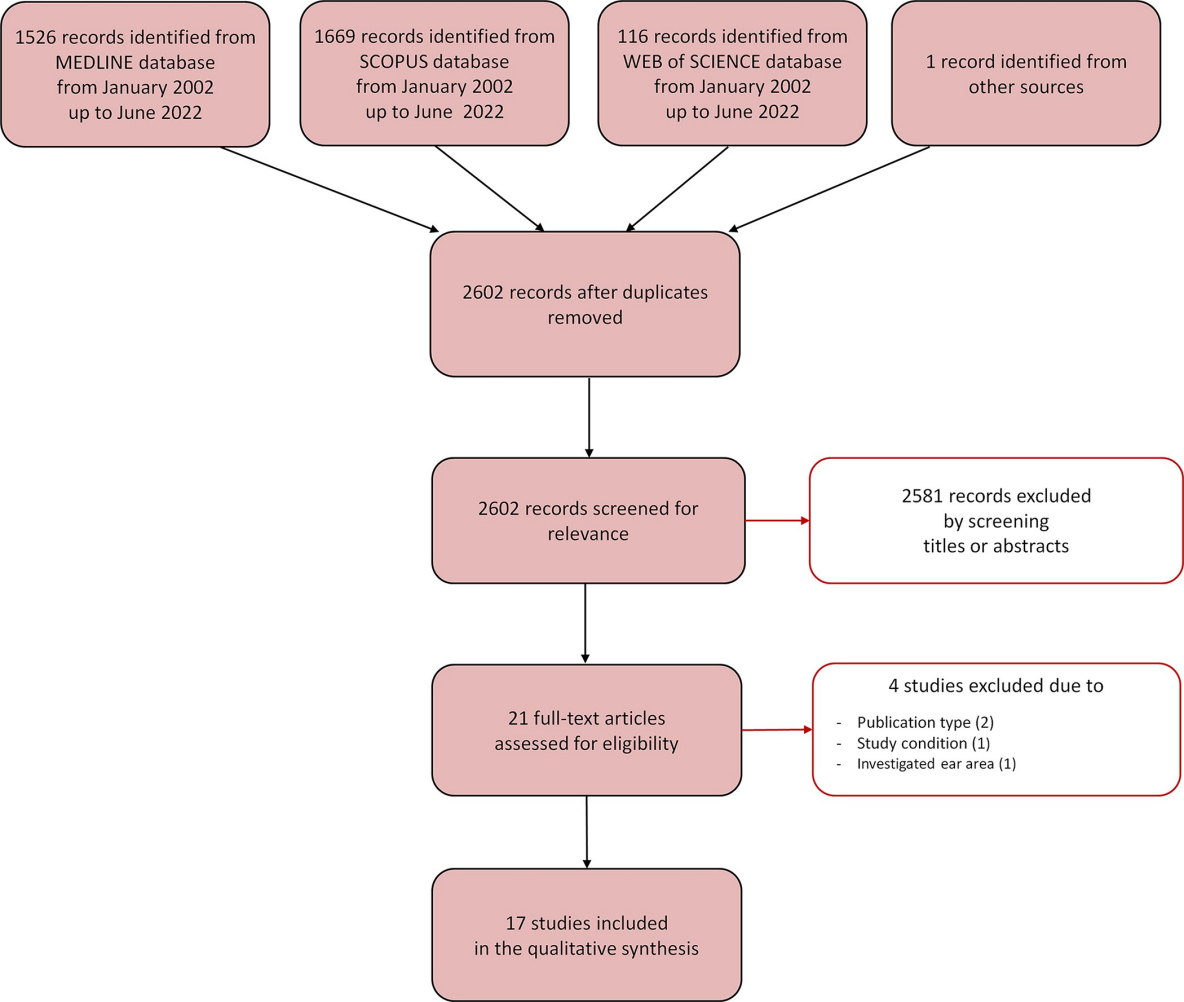
Selection process for the studies included in the scoping review. The Preferred Reporting Items for Systemic Reviews and Meta-Analyses (PRISMA-sc) flow diagram [31] depicts the number of records identified, included and excluded, and the reasons for exclusion, through the different phases of the scoping review.

The database search identified a total of 2602 relevant references once duplicates were removed (Fig 2). A total of 2581 references were excluded after reading title and abstract, and 21 were retrieved for further evaluation. Of these, four studies were excluded after reading thoroughly the text, because they did not fulfill the inclusion criteria, due to the publication type (i.e., two studies were reviews), the conditions of the study (i.e., one study was performed in sleeping instead of awake subjects), or the analysis of other ear regions (i.e., one study did not report data on the inner ear).

Following the selection process, 17 studies were included in the scoping review. Of these, nine studies evaluated the effects of HA exposure in a simulated setting and eight studies in a real HA environment. The studies are described in the next paragraphs and briefly summarized in Tables 2 and 3. The studies involved an overall population of 888 subjects, with single-study sample size ranging from six to 433 subjects (median of 15 subjects). The population was mainly composed of male subjects (88.7%) and the weighted average age was 27.8 ± 4.1 years (excluding one study where gender and age of the subjects were not reported [35]).

**Table 2:** Studies analyzing the effects of hypoxia on the inner ear function in a simulated environment. Studies are grouped based on the way high-altitude was simulated, i.e., as hypobaric hypoxia or normobaric hypoxia.

Study (Year)	Population	ENT technique	HA protocol	Results
***Hypobaric Hypoxia Setting***				
Lucertini et al. (2002) [14]	N = 6 (6 males) Age = 30 [25–37] y Normal hearing Acclimatization: NR	AER (SSR) Ear: right	• Altitudes: 5182 m in HH (2 exposures) and HN (after reoxygenation (100% O_2_)) MSL • Ascent rate: 12.3 m/s • Exposure duration: 53 min (two exposures separated by reoxygenation) • Measurement times: at MSL; at 6, 12, 18, 24, 30 min after arrival; at 6 and12 min after reoxygenation	• Significant increase in the latency of SSR sinus wave, without amplitude changes, 12 min after first HH exposure versus MSL, and 6 min after second HH exposure versus HN with reoxygenation (p<0.05). • Inverse correlation between latency and oxygen saturation levels (r = -0.58; p<0.000001).
McAnally et al. (2003) [36]	N = 10 (7 males) Age = 36.1 [23–46] y Acclimatization: NR	PTA Ear: right Note: stimuli calibrated at MSL	• Altitudes: 3700 m in HH and HN MSL • Ascent rate: 20 m/s • Exposure duration: 1 h • Measurement time: after 20 min at altitude	• Significant average threshold elevation of 2.57 dB (SE = 0.59, pooled among frequencies) between HH and HN (p = 0.002). • No significant frequency-dependent effect (p = NS), indicating no impairment of active processes.
Hayashi et al. (2005) [12]	N = 7 (7 males) Age = [20–26] Acclimatization: not acclimatized to 4500 m	AER (P300, N100) ABR (I, V waves, IV interpeak latency) Paradigm: active auditory oddball	• Altitudes: 4500 m in HH and HN (after reoxygenation (100% O_2_)) 610 m (baseline) • Ascent rate: 0.83 m/s • Exposure duration: 3 h • Measurement times: at baseline, at arrival, after 2h, 1 h after descent	• Significant increase of P300 latency, without changes in amplitude, at arrival to 4500 m vs baseline (p<0.01), with major prolongation after 2 h (p<0.0001). Return to baseline levels after reoxygenation (p<0.0004 vs 2h at 4500 m). • No significant changes in N100 throughout the study (P = NS). • Significant increase in ABR wave I and V peak latency and decrease in wave I amplitude at 4500 m vs baseline (p<0.0001). After reoxygenation, significant decrease in wave V latency vs no inhalation (p<0.0004), without significant changes in wave I amplitude nor wave I-V interpeak latency.
Ide et al. (2010) [13]	N = 6 (0 males) Age = 35.8 [29–49] y Normal hearing, no neurological disorders Acclimatization: NR	TEOAE DPOAE Ear: both Note: ear simulator to check non-physiological effects	• Altitudes: 5000 m in HH MSL • Ascent rate: 4.17 m/s • Exposure duration: 1 h • Measurement times: 20 min after arrival	• Significant reduction of TEOAE total echo-power by 4dB and of the SNR in the range 1.5–3.7 Hz in HH vs MSL (p<0.05), without significant changes in average reproducibility (p = NS). • Significant decrease in DPOAE between 1–6.5 Hz (except 1.3 Hz) in HH vs MSL (p<0.05). • Decrease of sound pressure stimulus of 3.8 dB in the ear simulator at 5000 m, suggesting a predominant non-physiological effect at the base of TEOAE and DPOAE reduction in HH.
Lucertini et al. (2020) [10]	N = 8 (8 males) Age = [27–53] y Normal hearing Acclimatization: fit for flight duties	PTA Ear: right Note: calibration correction for hypobaria artifacts on earphone	• Altitudes: 4600 m in HN (100% O_2_) and HH MSL • Ascent rate: NR • Exposure duration: 30 min • Measurement times: at MSL, in HN, and after 20 min in HH	• Significant difference in auditory threshold at 4000 Hz in HN (p<0.03) and HH (p<0.001) vs MSL for noncorrected data. Significance lost after correction (p = 0.08). • No difference between HH and HN for noncorrected nor corrected data.
***Normobaric Hypoxia Setting***				
Ogorodnikova et al. (2017) [37]	N = 33 (21 males) Age = [18–22] y No-cross over, subjects assigned to: • SSH (12), • IHT (11), • control (10) Acclimatization: NR	PTAEar: BothPsychophysical testing: Pause test, Rhythm test, Voice testTesting of auditory memory: short-term auditory memory test (Jacob test)	• Altitudes:5058–5249 m* in SSH or IHTMSL (control)• Ascent rate: immediate exposure• Exposure duration:SSH: 15 minIHT: sessions of 15 min over 1.5 months period• Measurement times: • PTA: at 5 min in SSH, before and after IHT, at MSL • Psychophysical testing: 5 min before and after SSH • Short-term auditory memory test: 5 min before and after SSH, before and after IHT	• Increase in auditory thresholds, especially for low frequencies, during SSH; but decrease in auditory thresholds, especially for high frequencies, after IHT. • Improvements in the qualitative and quantitative auditory analysis indicators, with a reduction in reaction times with SSH. • Improvements in auditory memory indicators, with an increase of reproduction productivity and volume of memory following SSH and IHT and major effects after IHT.
Seech et al. (2020) [38]	N = 40 (27 males) Age = 29.43 ± 5.99 y Normal hearing Acclimatization: NR	AER (MMN, P3a, RON) Paradigm: passive auditory oddball	• Altitudes 5442 m* in NH MSL (on separate day) • Ascent rate: immediate exposure • Exposure duration: 27 min • Measurement times: along 27 min in NH	• Significant reduction in P3a amplitude with hypoxia (p = 0.0001) and time (p<0.01). • Significant effect of time (p = 0.02) on RON amplitude. • No effect on MMN amplitude.
Blacker et al. (2021) [39]	N = 26 (14 males) Age = 30.54 ± 6.59 y Normal hearing, no psychological or neurological disorders Acclimatization: NR	AER (MMN, P3a) Paradigm: passive auditory oddball	• Altitudes: 6049 m* in NH MSL (at 21% and 100% O_2_, baseline) • Ascent rate: immediate exposure • Exposure duration: 10 min Measurement times: at baseline, during 10 min at HA, and during 4h of recovery (at 21% and 100% O_2_)	• Significant reduction in MMN amplitude in NH and till 60 min after exposure vs baseline (p<0.05) and significantly shorter peak latencies from 20 till 180 min after exposure (p<0.05). • P3a transiently affected, with higher amplitude only in NH vs baseline (p<0.05) and no effect on latency (p = NS).
Blacker et al. (2022) [2]	N = 34 (16 males) Age = 29.12 ± 6.19 y Normal hearing, no psychological nor neurological disorders Acclimatization: NR	AER (P50, N100, P200, gating ratios (S1/S2) and gating differences (S1-S2)) Paradigm: paired-clicks condition-test	• Altitudes:6049 m* in NHMSL (on separate day) • Ascent rate: immediate exposure • Exposure duration: 14.5 min • Measurement times: at SL, during 14.5 min at HA	• Significant stimulus x condition effect (p = 0.01) on P200, indicating a diminished sensory gating effect during NH vs MSL.

*, calculated from effective oxygen percentages according to standard conversion charts. Data are expressed as numbers, mean ± SD, median [range], or [range], as available. Ascent rates were always expressed in meters/seconds (m/s) according to standard transformations. ABR, auditory brainstem response; AER, auditory-evoked response; DPOAE, distortion product oto-acoustic emissions; h, hour; HA, high altitude; HH, hypobaric hypoxia; HN, hypobaric normoxia; IHT, intermittent hypoxia training; min, minutes; MMN, mismatch negativity component; MSL, mean sea level; NH, normobaric hypoxia; NS, not significant; NR, not reported; O_2_, oxygen; p, p-value; PTA, pure-tone audiometry; r, Spearman’s correlation coefficient; RON, re-orientating negativity; SE, standard error; SNR, signal-to-noise-ratio; SSH, single session hypoxia; SSR, steady-state response; TEOAE, transient evoked oto-acustic emissions; TTS, transient threshold shift; vs = versus; y, years.

**Table 3:** Studies analyzing the effects of hypoxia on the inner ear function in a real in-field environment.

Study (Year)	Population	ENT technique	HA protocol	Results
Singh et al. (2003) [40]	N = 15 (15 males) Age = [21–25] y Acclimatization: no previous HA exposure	AER (N1, P2, N2, P3) Paradigm: standard auditory oddball	• Altitudes: 3500 m (Western Himalayas) MSL (Delhi) • Exposure pattern and duration: 1 month Measurement times: at MSL, 1^st^ and 3^rd^ week at 3500 m, return to MSL	• Significant increase in P3 wave latency in the first (p<0.05) but not in the third week (p = NS) vs MSL. Non-significant variations in P2 and P3 amplitude were found during 1^st^ and 3^rd^ week of recording at HA (p = NS). • No significant difference in AER between values at MSL before and after the expedition (p = NS).
Singh et al. (2004) [16]	N = 20 (20 males) Age: 25 [21–32] y Acclimatization: no previous HA exposure	AER (N1, P2, N2, P3) Paradigm: standard auditory oddball	• Altitudes: 3200 m (Kyangnosla, Eastern Sikkim) 4300 m (Nathula, Eastern Sikkim) MSL • Exposure pattern and duration: 2 weeks (6 days at 3200 m and 8 days at 4300 m) • Measurement times: at MSL, after 6 days at 3200 m, after 8 days at 4300m, at return to MSL	• Significant increase in P3 wave latency at 3200 m (p<0.01) and 4300 m (p<0.05) vs MSL. Slight increase in P2 and P3 wave amplitude at 3200 m and 4300 m compared to MSL (p = NS). • Latencies of N1, P2, N2, P3 near to sea level values on return from HA.
Singh et al. (2004) [15]	N = 30 (30 males) Age: [21–32] y Acclimatization: no previous HA exposure	ABR (peak latency of wave I, III, and V, interpeak latency of I–III, III–V and I–V). Paradigm: monoaural auditory click stimuli	• Altitudes: 3200 m (Eastern Himalayas) 4300 m (Eastern Himalayas) MSL • Exposure pattern and duration: 2 weeks (6 days at 3200 m and 8 days at 4300 m) Measurement times: at MSL, at 4^th^ day at 3200 m and 4300 m, at return to MSL	• Significant increase in wave I and wave III peak latencies at 4300 m vs MSL (p<0.05 and p<0.01, respectively), and in peak V latency at 3200 m vs MSL (p<0.05). • On return to MSL, wave III peak latency still significantly higher than at MSL before ascent (p<0.01).
Olzowy et al. (2008) [41]	N = 13 (13 males) Age: [28–65] y Acclimatization: NR	DPOAE Ear: NR Note: DPOAE levels at 500 m taken as baseline for every climber and subtracted from DPOAE levels at HA	• Altitudes: 500, 2500, 5100 (Base Camp), 6000 m (Camp I) 6900 m (Camp II, 7 climbers) 7400 m (Camp III, 6 climbers) during ascent to Gasherbrum II (Karakorum) • Exposure pattern and duration: 36 days of expedition, 15 days to reach the Base Camp, 20 days at HA ≥ 5100 m • Measurement times: when reaching each indicated altitude	• Significant changes in DPOAE at the highest altitude reached vs 500 m for frequencies of 1, 3 and 4 kHz (P<0.001). • No significant changes in DPOAE at 2500 m vs 500 m (p = NS).
Olzowy et al. (2014) [35]	N = 187 (gender NR) Age: NR Acclimatization: no previous exposure HA	DPOAE Ear: NR Note: DPOAE levels at 500 m taken as baseline for every climber and subtracted from DPOAE levels at HA.	• Altitudes: 2610 m (Phakding, Mount Everest) 5170 m (Gorak Shep, Mount Everest) • Exposure pattern and duration: Mount Everest Trek, no details reported • Measurement times: at trekking start at 2610 m and when reaching 5170 m	• Significant decrease in DPOAE levels at all measured frequencies at 5170 m vs 2610 m (p<0.05), without dependence on AMS development nor LLS. Significant but weak correlation between the extent of DPOAE decrease and oxygen saturation decrease at 1 kHz (R^2^ = 0.027, p = 0.046).
Neri et al. (2014) [42]	N = 7 (7 males) Age = 39.4 [22–60] y Acclimatization: NR	PTA DPOAE Ear: both	• Altitudes: 5000 m (Manaslu Base Camp) 5900 m (Camp 1) 6400 m (Camp 2) MSL • Exposure pattern duration: 21 days at base camp (25 km/day (7 h per day) for reaching Base Camp); ascents to Camp 1 and 2 with favorable conditions (8–10 km/day (6 h per day) to reach Camp 1 and 2) Measurements times: at MSL, before and after the expedition	• PTA values in the normal range without significant differences before vs after expedition (p = NS). • DPOAE stable in repeated measurements of the same ear before vs after expedition (p = NS).
Saini et al. (2021) [43]	N = 433 (433 males) Age = 27.13 y Acclimatization: NR	PTA Ear: both	• Altitudes: [3048–3658] m • Exposure duration: > 1 year • Measurement times: at HA induction, and after 1 year of HA	• Significant biaural increase of hearing thresholds (p<0.05) at all frequencies, with maximum worsening at 4000 Hz in the right ear. • Worsening in four-tone average of pure-tone thresholds by 3.08% in the left ear vs 64% in the right ear.
Fehrenbacher et al. (2021) [11]	N = 13 (4 males) # Age: [22–29] y Acclimatization: NR	PTA TTS (white noise of 90 dB for 10 min between audiometric exams). Ear: both Note: sound level of white noise adjusted according to altitude	• Altitudes: 5300 m (Ghorak Shep) 1400 m (Kathmandu) • Exposure pattern and duration: ADEMED expedition 2011, 34 days of expedition, 10 days to reach 5300 m, 15 days at 5300 m • Measurement times: at 1400 m, when reaching 5300 m after 10 days of trek, at return to 1400 m	• Significant difference in TTS between 5300 m vs 1400 m both before (p<0.0001) and after (p = 0.0014) expedition. • Non-significant difference in TTS at 1400 m before vs after the expedition (p = NS).

#, one subject of 52 years was excluded from the analysis due to a pre-existing dip at 4000 Hz. Data are expressed as numbers, mean ± SD, median [range], or [range], as available. ABR, auditory brainstem response; AER, auditory-evoked response; AMS, acute mountain sickness; DPOAE, distortion product oto-acoustic emissions; HA, high altitude; LLS, Lake Louise Score; MSL, mean sea level; NR, not reported; NS, not-significant; p, p-value; PTA, pure-tone audiometry; R^2^, Pearson’s correlation coefficient; TTS, transient threshold shift; NS, not significant; vs = versus; y, years.

### 3.2 Studies in simulated environment

The nine studies evaluating the effects of hypoxia on the inner auditory function in a simulated environment are shown in Table 2. In five studies conditions of HH were simulated, while the remaining four studies were performed under NH. Three studies (two in HH [10, 36] and one in NH [37]) used PTA, one study in HH analyzed OAE [13], and five studies (two in HH [12, 14] and three in NH [2, 38, 39]) analyzed AER. All these studies had an exposure time of less than six hours.

Considering the studies performing PTA, McAnally et al. [36] studied 10 healthy subjects at mean sea level (MSL) and during acute exposure (one hour) to an equivalent of 3700 m, either with (i.e., hypobaric normoxia [HN]) or without oxygen supply by a mask (i.e., HH). The specific effects of the hypoxia on hearing function were estimated by calculating the difference in the measured thresholds between the mask-on and mask-off conditions at MSL and subtracting it from the difference measured at simulated 3700 m. The authors observed that hypoxia induced a significant (p<0.002) elevation of auditory threshold of 2.57 dB on average, without frequency-specific effects (p = 0.75, i.e., all frequencies seemed similarly affected). Lucertini et al. [10] assessed PTA in 8 male subjects at MSL and during acute exposure (half an hour) to a simulated altitude of 4600 m, with the subjects breathing 100% oxygen or air. The authors evaluated the effects of correcting measurements in hypobaria according to a recalibration table, which considered the artifactual contribute of the lower air pressure in sound wave transmission and transduction on the headphone parts. A significant reduction of ear sensitivity (i.e., increase of the hearing threshold) was detected at the 4000 Hz frequency in both HH (p<0.001) and HN (p<0.03) versus MSL for non-corrected data, but statistical significance was lost (p = 0.08) when corrected data were considered. Ogorodnikova et al. analyzed the effects of NH on PTA as well as on psychophysical properties of auditory analysis and short auditory memory [37]. In the study, 33 healthy subjects were alternatively exposed to control conditions, a single 15 min session of NH at 10.9–11.2% oxygen concentration (~5000 m), or to sessions of interval hypoxic training (IHT) over a period of 1.5 months. During single-session hypoxia (SSH) exposure, participants experienced an increase in auditory thresholds of 9 dB maximum, especially at low frequencies (up to 500 Hz), but differences did not reach statistical significance (p = NS). After IHT, the subjects displayed instead an increase in auditory acuity (i.e., a decrease in hearing threshold), especially for high frequencies.

Only one study analyzed TEOAE and DPOAE under HH conditions [13] by exposing six female subjects to a simulated altitude of 5000 m (one hour). Variations in the sound pressure stimulus with altitude were also evaluated on an ear simulator to assess potential confounding effects. TEOAE total echo power was 4 dB lower (p<0.05), and signal-to-noise ratio was significantly (p<0.05) reduced in the range 1.5–3.7 Hz in HH compared with the measurements at MSL. Similarly, in HH condition, DPOAE were significantly lower than at MSL at frequencies between 1 and 6.5 kHz (p<0.05). Measurements on the ear simulator showed a decrease in sound pressure stimulus and sound pressure readings for the microphone (especially for higher frequencies) at the decrease of air pressure. The decrease was of the same entity of the changes observed in OAE.

Focusing on studies analyzing EEG-based measurements, Lucertini et al. investigated auditory SSR in six male healthy volunteers at MSL and during a 30 min stay at a simulated altitude of ~5200 m [14]. Auditory SSR was also measured at altitude after two minutes of re-oxygenation, when the mask was taken off. An increase in the SSR wave latency was observed during hypoxia, with changes becoming significant (p<0.05 versus MSL) after 12 minutes of exposure. During re-oxygenation, the latency got back to basal values, but it increased significantly within six minutes from the beginning of the second HH exposure (p<0.05 versus reoxygenation). A significant inverse correlation (r = -0.58; p<0.000001) was observed between P1 latency and oxygen saturation values among subjects, with an increase in the latency at the decrease of saturation. Conversely, no changes in wave amplitude were observed. Hayashi et al. studied ABR and late AER in seven male subjects after a rapid ascent from an acclimatization altitude of 610 m to a simulated altitude of 4500 m [12]. Measurements at altitude were performed after arrival, after two hours of permanence at altitude, and after 100% oxygen inhalation to regain basal oxygen saturation values. A significant change in ABR was observed with altitude, where the latency of wave I and V peaks increased and wave I amplitude decreased at altitude (p<0.0001). Evaluation of late AER showed no change in N100 with altitude, and a prolongation of P300 latency after two hours of exposure, without significant changes in the amplitude. Oxygen administration brought P300 latency to baseline values. A series of studies from Blacker’s group [2, 38, 39] investigated the effects of NH on AER that are associated with different stages of auditory sensory processing. Seech et al. (2020) investigated auditory deviance response (early sensory processing) to an auditory oddball paradigm by analyzing cognitive event-related potentials, such as mismatch negativity (MMN, peak at 120–200 ms), P3a (peak at 250–320 ms), and re-orientating negativity (RON, peak at 350–450 ms), which are evoked in response to unattended changes in repetitive background stimulation [38]. Forty subjects were tested at MSL and within the first 27 min of exposure to NH (equivalent to ~ 5400 m). While no significant changes were observed for MMN and RON after exposure, P3a amplitude was significantly decreased (p = 0.0001) within the first nine minutes of exposure and further decreased over time (p<0.01). In a subsequent study [39], the authors investigated the recovery of auditory processing after acute exposure to a higher simulated altitude (~ 6000 m) over a four-hour window and compared the effects of different recovering breathing gases (21% or 100% oxygen) on performance. MMN showed an attenuated amplitude at NH versus baseline (the effect persisting till one hour after exposure (p<0.05)) and a decreased latency in hypoxia (persisting up to three hours after exposure (p<0.05)). Conversely, the amplitude of P3a was only transiently affected by NH exposure, showing an increased amplitude (p<0.05) without significant changes in latency and returning to baseline level immediately after the exposure. In both cases, no significant effect of the recovery gas was observed. The results on “cognitive” event-related potentials [38, 39] were recently integrated by analyzing hypoxia effects on early long-latency AER, related to sensory processing and gating (P50 and N100) or beyond sensory perception (P200), using a two-click paradigm [2]. The sensory gating ratio for auditory stimuli was intact for paired responses of the P50 and N100. However, P200 sensory gating ratio was significantly (p = 0.01) attenuated under NH compared to normoxic conditions.

### 3.3 Studies in in-field environment

The eight studies evaluating the effects of HA on the inner auditory function in a real environment are shown in Table 3. Among these studies, three performed PTA [11, 42, 43], three characterized OAE [35, 41, 42], and three analyzed AER [15, 16, 40]. Only one study analyzed the effects of a chronic exposure at HA [43], while all the others investigated a pattern of acute exposure to HA with a period of acclimatization involving physical exercise.

Considering PTA evaluation, Saini et al. analyzed the effects of chronic exposure to HA by studying both ears in 433 lowlanders at initial exposure and after one year of permanence at altitudes higher than 3000 m [43]. A significant worsening (p<0.05) was found in both ears for all frequencies in the range 500–4000 Hz. The decline in performance was maximum at 4000 Hz, especially in the right ear, where the four-tone average of pure-tone thresholds worsened by 64%.

Focusing on acute HA exposure, Neri and colleagues did not find any significant difference in PTA measurements performed in seven subjects before and after an expedition to a maximal altitude of 6400 m [42]. Fehrenbacher et al. investigated the combined effect of noise and HA on hearing function by performing PTA before and after white noise exposure to induce temporary threshold shift (TTS), at 1400 m (Kathmandu), 5300 m (Ghorak Shep), and at return to 1400 m after HA exposure [11]. A significant increase in mean TTS was observed at Ghorak Shep versus Kathmandu, either before and after exposure (p<0.0001 and p = 0.0014, respectively).

Regarding OAE measurements, DPOAEs were evaluated by Olzowy and co-workers at altitudes of 500 (baseline), 2.500, 5.100, 6.000, 6.900 and 7.400 m, in 13 healthy males during a 36 day ascent to 8035 m (Gasherbrum II) in the Karakorum [41]. Significant changes (p<0.001) in DPOAE levels at the frequency of 1, 3, and 4 kHz were observed between baseline and the highest altitude reached by the climbers (> 6000 m). One climber developed severe AMS with the most pronounced decline of DPOAEs at 1 kHz. No significant changes were found in DPOAE levels between measurements at 500 m and those at 2500 m. In a second study by the same group [35], DPOAE measurements were taken in a larger population of 187 climbers, who ascended from 2610 m to 5170 m in the Mount Everest trek. A significant decrease in DPOAE level between the two altitudes was found at all frequencies, but the decrease displayed a significant correlation (R^2^ = 0.027, p = 0.046) with oxygen saturation values only at 1 kHz. DPOAE levels decreased at altitude at all frequencies without a difference between trekkers with AMS and without AMS. DPOAE were evaluated also by Neri et al. in seven subjects returning from an expedition to 6400 m [42]. During permanence at base camp, AMS symptoms were insignificant in all climbers, and none needed any medication. Repeated measurements of the same ear were performed before and after the expedition and the comparison pointed out the absence of DPOAE alteration when subjects were back at MSL (p = NS).

Focusing on AER characterization, three studies were performed by the same group during different expeditions. In the first study by Singh et al., 15 healthy males underwent AER recording with a standard auditory odd ball paradigm, at MSL in Delhi and at during a one-month period at 3500 m in the Western Himalayas [40]. A significant increase (p<0.05) in the latency of P3 wave was detected during the first week at HA with respect to MSL, while the increase did not reach statistical significance after three weeks at HA. In a subsequent study [16], AER changes at HA were evaluated in 20 healthy males at MSL and during a two-week expedition in the Eastern Sikkim. Like previous results, P3 wave latency resulted significantly increased after six days at 3200 m (p<0.05) and after eight days at 4300 m (p<0.01) with respect to MSL, and it returned almost to MSL values after return from HA. In a third study, ABR were recorded in 30 healthy males during a two-week expedition in the Eastern Himalayas [15]. The recording evidenced a significant increase in the peak latency of wave I (p<0.05) and III and (p<0.01) on the fourth day at 4300 m and in the peak latency of wave V on the fourth day at 3200 m (p<0.05) with respect to MSL. The peak latency of wave III remained significantly higher (p<0.01) even after returning to MSL.

## 4. Discussion

In this scoping review we collected and analyzed all the available evidence from the last 20 years on the effects of HA exposure on the auditory function. We identified 17 studies, which mostly observed auditory function alterations at HA, although HA effects displayed variable magnitude and specific features. This variability in auditory outcomes may be ascribed to the significant heterogeneity observed among studies in terms of HA exposure protocols, as well as in terms of the techniques used to assess auditory function, which needs to be considered for a critical appraisal and comparison of the results. As concerns HA exposure protocols, studies comprised either simulated and in-field conditions. In simulated studies, HA was reproduced in terms of a sole reduction of inhaled oxygen (i.e., NH) or including the effect of atmospheric pressure changes (i.e., HH), where the additional condition of hypobaria may exert physical alterations in sound transmission [44] and produce more severe physiological effects than hypoxia alone [3–5]. In all the simulated cases the subjects had an exposure time of less than six hour with a high speed of ascent (above 4 m/min in all except one). In the in-field studies, subjects were exposed to altitude for a longer period (from two-weeks to one year). Among in-field studies, only one study considered the effects of chronic HA exposures [43], while the remaining studies considered subjects during trekking. Both simulated and in-field conditions presented differences in the maximum altitude reached (ranging from 3700 m to 6049 m in simulated and from 3500 m to 7400 m for in-field studies). Differences in the spatio-temporal profile of HA exposure may determine significant differences in the involved regulatory mechanisms and acclimatization processes and thus in the observed physiological effects. Considering that PTA, AER, and OAE assess different physiological features and anatomical pathways of auditory function, results are discussed below according to the nature of the auditory testing performed in simulated and in-field conditions. Together with the variability of the protocols, a limitation affecting several studies included in the present review is the small sample size of the analyzed population, where nine of 17 studies presented a study population equal to or less than 15 subjects. This factor, combined with large inter-individual variability related to demographic and physiological aspects (e.g., variability in oxygen transfer system, chemoreceptor sensitivity, respiratory activity, and hypoxia-induced hyperventilation [14, 16]), may explain variable results among studies or non-significance of changes (e.g., for AER amplitude). Demographic variability could be partially tackled by restricting enrollment to selected population (e.g., military aviators or trekkers), although this may limit the extension of the results to the general population.

### 4.1 Evaluation of PTA at HA

The six studies measuring PTA in this scoping review [10, 11, 36, 37, 42, 43] almost consistently showed that HA induced alterations in hearing thresholds, although with differences in magnitude and frequency-dependence. Among variability factors, the six studies included either simulated [10, 36, 37] and real HA conditions [11, 42, 43], with consequent differences in the exposure duration, magnitude, and in the pattern of exposure (e.g., single sessions versus intermittent hypoxia). Focusing on in-field studies [43], prolonged exposure (1 year) to an altitude higher than 3000 m induced a statistically significant deterioration in hearing thresholds at frequencies in the range 500Hz—4 kHz, with one third of participants showing an hearing threshold augment > 5 dB. Similarly, exposure of shorter duration (10 days) at more pronounced HA (5330 m) [11] exacerbated the effects of noise exposure on hearing threshold, resulting in an amplified TTS. In contrast, only a slight, but not significant, decrease in hearing capacity was reported when comparing measurements before and after a three-week expedition at > 5000 m [42]. Although the absence of significant results may be partially attributed to the small sample size of the study, it may also indicate the short-term transient nature of alterations induced by HA. While in real settings the effects of hypoxia could not be separated from those of hypobaria, in simulated settings the two mechanisms were analyzed comparing conditions of HH, NH, and HN. However, different studies applied diverse compensation measures to control for the effects of hypobaria, which should be considered when comparing measurements. The reduction of air pressure at altitude is known to exert physical effects on sound transmission and on PTA instruments (microphones and earphones). The transfer function of electro-mechano-acoustical components of the earphone was shown to vary with altitude and the output peak of the system to progressively shift to lower frequencies at higher altitudes, determining frequency-specific changes of the stimulus intensity and relevant variations of the final sound pressure level recorded by the sound level meter [44]. To control for these physical artifacts, Lucertini et al. analyzed the difference between the sound pressure level recorded at several altitude levels (from around 2700 to 10700 m) with respect to sea level in an artificial ear system, determining frequency-selective and altitude-related stimulus correction factors for recalibrating the earphones’ output [44]. In [10], the authors applied these correction factors to analyze audiometric data collected in HH and HN conditions at 5000 m with respect to MSL. An elevation of hearing thresholds at HA with respect to MSL was observed at 4000 Hz for both HH and NH, without significant differences between the two conditions. However, threshold elevation was statistically significant only for uncorrected data. Based on these results, the authors suggested the crucial influence of hypobaria *per se* in sound transmission and loudspeakers performance at HA and pointed out the importance of performing appropriate correction for physical effects when comparing results obtained at HA with those at ground level. Beyond the physical effects of low barometric pressure on acoustic wave propagation, hypobaria may also exert physiological effects. HH is considered to have more severe impact than NH, the former being characterized by lower oxygen saturation, minute ventilation, alveolar ventilation, and tidal volume, increased dead space, hypocapnia, and alkalosis, and altered alveolo-capillary diffusion [3–5]. Alterations in hypoxia and hypocapnia directly impact cerebral blood flow and thus hearing function. Differences in physiological responses to HH and NH should also be considered, in addition to physical effects of hypobaria, to isolate hypoxia effects. A separation approach different from Lucertini 2020 [10] was applied in [36], where the specific effects of hypoxia were calculated by subtracting the effects of HN to those of HH at simulated 3700 m and MSL, yielding an average elevation of the hearing threshold of 2.57 dB in hypoxia, without significant frequency-dependence effects. Of note, the subtraction approach assumes that the effects of hypobaria and hypoxia are mainly additive. However, given the network of physiological mechanisms acting and interacting in the presence of the two conditions, the approach may be oversimplified to identify “pure” hypoxic effects. Although the available evidence does not allow to perform a punctual comparison of HH versus NH effects on PTA, the studies in the present review consistently reported an increase in hearing threshold in both conditions. The sole exception was the condition of IHT in NH in [37], where the authors reported a decrease of hearing threshold and a tendency toward improved auditory sensitivity. However, this result is likely related to the spatio-temporal pattern of IHT instead of to the normobaric condition, since normobaric SSH in the same study produced an increase (albeit non-significant) in auditory threshold.

Hypoxia-related alterations in hearing threshold shifts detected by PTA may be explained in the framework of hemodynamics and hemoconcentration changes at HA [45]. Oxygen supply to the ear occurs through diffusion over the round window and/or the blood [17] or through the endolymph [46] and oxygen must diffuse over a long distance from the vessels in the limbus spiralis and from the perilymph of the scala tympani [18]. This specific vasculature may accentuate the effects of arterial oxygen decrease limiting oxygen supply to the inner ear and potentially compromising hair cells functionality and integrity [11]. Additional environmental conditions, such as the presence of low barometric pressure (i.e., HH versus NH), may exert a higher impact on cerebral than skeletal muscle oxygen delivery [3], potentially due to blunted cerebrovascular reactivity, which may lead to a further decrease in oxygenation to the brain and thus to the ear. Fehrenbacher and colleagues hypothesized that the hypoxia-related increase in TTS and the eventual induction of permanent threshold shifts could be attributed to an increased ATP request to support cochlear cell function, not compensated by an adequate increase of oxygen supply [11]. Other authors hypothesized that the uniform loss in hearing sensitivity observed at HA might have been instead related to a change in endocochlear potential, which acts as the “battery” of the hair-cell receptor potential [36]. On the other hand, positive effects of hypoxia on hearing function, as observed during IHT in contrast to SSH [37], may be associated to long-term adaptive rearrangements and activation of body’s protective reserves [37]. All these hypotheses however need further experimental validation.

### 4.2 Evaluation of OAE at HA

The studies focusing on OAE analysis in HA conditions have generally found a reduction in OAE intensity during or after exposure [13, 35, 41, 42]. However, such findings must be cautiously interpreted due to technical challenges and uncertainty related to the mechanisms underlying OAE decrease.

Ide et al. found a significant decrease in both TEOAE and DPOAE values after hypobaric hypoxia in a simulated environment [13]. Such results could suggest a rapid decrease in cochlear activity, since the experiment was performed after a 30-min exposure to low pressure and oxygen saturation. However, when the same experiment was performed with an ear simulator, it was noted that the reduction in air pressure affected, as expected, stimulus sound pressure levels. A global decrease in the intensity of both the stimulus and the evoked response could be due to non-physiological factors and must be adjusted for when performing OAE measurements at low air pressure [13]. This technical problem can be mitigated by modern equipment that is able to adjust sound output in order to maintain the same level of intensity [44]. However, it is also important to expect the backwards-traveling sound wave coming from the cochlear amplifier to travel in the same low-pressure medium. So it can be postulated that, in hypobaric conditions, globally reduced OAE levels do not inherently reflect a reduction in cochlear functionality [44]. In addition to this, it must also be considered that the shape and length of the external auditory canal, as well as the depth of the probe insertion, may alter the signal-to-noise ratio in a significant way. These artifactual effects must be accounted for with the use of filtering and calibration [44], which are available in modern OAE software, but can offer additional challenges for in-field OAE measurements, such as the excessive amount of time required.

In-field studies conducted by Olzowy et al. [35, 41] showed a decrease in DPOAE levels at altitudes exceeding 5100 m, with respect to baseline (500 m). However, such alterations in DPOAE did not always reflect the entity of hypoxic injury as measured by oxygen saturation levels. Authors postulated that a threshold for hypoxic damage to cochlear function may exist, above which significant changes in DPOAE may go unnoticed [35, 41]. Another possible reason suggested by this research group for a decrease in DPOAE levels is a change in intracranial pressure (ICP). Several studies have linked an increase in ICP to lower DPOAE levels, thus suggesting OAE measurements as a non-invasive tool to measure ICP in subjects needing constant monitoring for sudden shift in cerebrospinal fluid pressure, such as astronauts or fighter pilots [47]. The physiological rationale of this hypothesis consists in the direct connection between cerebrospinal fluid and the inner ear fluids via the cochlear aqueduct, which in turn can impair the movement of inner ear membranes and middle ear structures, thus changing the mechanics of the system and altering the shape and characteristics of the outbound OAE waveforms [48]. However, some limitations and pitfalls exist. It is still not possible to correlate the extent of ICP increase to DPOAE disruption. It is also necessary, due to the nature of DPOAE signals having high inter-individual variability, to have a baseline measurement for any individual subject, and finally, DPOAE impairment observed in conditions of elevated ICP frequently impact lower frequencies, where signal-to-noise ratio is lower [47].

Olzowy et al. was one of the few groups to evaluate a possible link between DPOAE and AMS, based both on the debated relationship between AMS and HA cerebral edema and a rise in ICP [7]. The clinical observation from their first study suggested that the single climber developing severe AMS had the most pronounced decline of DPOAEs at 1 kHz [41]. However, in a further study the authors reported that DPOAE levels decreased at all frequencies at altitude without a difference between trekkers with AMS and without AMS. It is very likely that other components than ICP might concur in causing a decrease in cochlear function as measured by OAEs, with reduction in vascular support to the inner ear and blood hyper-osmolarity representing candidates, which merit further investigation [35].

In contrast with previously cited works, results by Neri et al. showed no significant changes in DPOAE levels after a mountain expedition reaching an altitude of 6400 m [42]. However, measurements were recorded before and after such expedition, so that it is possible that subtle changes in inner ear dynamics and in cochlear function could already have reversed to physiological conditions at the time the evaluation was performed.

### 4.3 Evaluation of AER at HA

Eight studies [2, 12, 14–16, 38–40] were identified in the present scoping review that analyzed auditory function in simulated and real HA conditions by means of EEG-derived measurements. These studies pointed out hypoxia-induced slowing of acoustic signal transmission and elaboration at different levels of the auditory pathways, although with some variability among studies. In addition to the aforementioned variability factors related to HA protocols, difference in responses when performing cognitive tasks may be attributed to different arousal levels and subject-specific extra effort for task performance under HA conditions [16]. Lack of evidence emerged regarding long-term exposure and acclimatization processes on AER, where the only available observations came from one single group in a series of studies conducted 20 years ago [15, 16, 40].

Studies in both simulated and in-field conditions were quite consistent in indicating an increased response latency, while data on amplitude were sparser. Latency changes should mainly indicate a prolonged impulse transmission time at the level of synapses, while amplitude variations may suggest a decreased synchronism in neural discharge [14]. Considering indicators of sound transmission from the cochlea to the brainstem and subcortical areas, such ABR, increased peak latency for wave I and V were observed in simulated HH conditions at a altitude of 4500 m, accompanied by a decrease of wave I amplitude [12]. In-field study results confirmed a significant increase in peak latency of wave I and III at 4300 m and of wave V at 3200 m versus MSL, with the effects on wave III persisting after exposure [15]. As suggested by Singh et al. [16], ABR peak latency and its variability may be affected by hyperventilation leading to hypocapnia and respiratory alkalosis through the dampening effect on the ascending reticular activation system.

Results about late AER and auditory SSR, reflecting activity from higher brain regions showed attenuated responses in hypoxia for exogenous and endogenous potentials as well as for SSR. In in-field conditions a significant increase in P3 wave latency was found at 3500 m [40] and 4300 m [16] versus MSL, while a non-significant latency increase at 4300 m was found for N2 wave [16]. In simulated studies, a significant reduction in P3a amplitude at ~ 5400 m was observed during a passive auditory oddball paradigm in conjunction with a continuous visuomotor tracking task [38]. However, P3a amplitude was only transiently affected by exposure to ~ 6000 m [39], while major effects, such as attenuated amplitude and decreased latency, were observed on MMN potential. The sensory gating ratio for paired response of P200 (but not of P50 and N100) was significantly reduced at simulated 6000 m [39]. Endogenous potential P300 evoked by oddball paradigm showed maximal latency prolongation, without amplitude changes, after two hours at 4500 m, which was compensated by oxygen inhalation [12]. SSR wave latency was found to increase in parallel with hypoxia with significant increase in P1 latency and an inverse correlation between latency and oxygen saturation values at simulated HH of >5000 m [14]. Changes became significant already after 12 and 6 minutes following the first and second HH exposures, respectively, and were reversed by reoxygenation [14]. Comparing the effects of HH and NH conditions on AER, studies in simulated HH [12] and in-field [16, 40] similarly showed an increase in the latency of P3/P300 wave with only slight or no change in amplitude, while studies in NH showed some alterations although not consistent (reduction in [38], increase in [39]) in P3a amplitude without changes in latency. Although these differences might be in part related to the minor impact of NH with respect to HH, the comparison is hindered by the small number of studies and by the presence of additional differences in protocols (e.g., diverse cognitive test protocols).

Different physiological mechanisms may underlie hypoxia-related changes in AER [2], including peripheral (cochlear) and central dysfunction. Considering peripheral mechanisms, a depression of endocochlear potential may underlie the changes in the latency and amplitude of wave I, which are suggestive of an increase in ABR threshold [49]. Hypoxia-related alterations at the cochlear level would be consistent with the reduction of cochlear function suggested by OAE measurements [22, 25], as well as with the hypothesis that PTA threshold augmentation may be related to changes in endocochlear potential [11, 36]. The increase in ABR threshold would lead to a decrease in auditory stimulus intensity that may exert effects also on the superior levels of the auditory pathways, affecting each AER component. It may explain the prolongation of peak latency observed also for III-V waves [12, 15, 16, 40] and concur with central mechanisms to the alterations of AER components related to higher processing [12], such as “cognitive” AER [12, 39] and “sensory” AER [50]. As concerns central mechanisms, hypoxia may alter brainstem neuron response [12] and induce changes in the synthesis and/or release of specific neurotransmitters, such as acetylcholine, which may contribute to cognitive performance alterations [51]. A decreased sensitivity of the brainstem neurons to auditory input, a decrease in the signal to the auditory cortex, and a decrease in cognitive processing speed [2] may contribute to the lengthening of AER latency consistently observed among studies. The increased latency of potentials related to cognitive processing of a stimulus [16], such as N2 and P3, at HA may indicate an effect of hypoxia on cognitive function and may directly affect the absolute timing of decision processes in sensory discrimination. Focusing on potentials, such as N100 and P200, related to higher brain regions and early sensory gating functions for triggering and allocating attention [52], the absence of changes in N100 under hypobaric/hypoxic conditions may indicate the preservation of cortical function related to early attention and stimulus processing, while the reduction in sensory gating of P200 may point out an impaired allocation of auditory attention in hypoxia [39]. The influence of acute hypoxia on auditory information processing at the attention level would be supported also by the reduced amplitude of MMN [39] and P3a [38] at HA, where the MMN/P3a response complex is related the detection of and attention orientation for a deviant stimulus [53]. The prolongation of P300 latency at HA may indicate a deterioration of cognitive activities involved in stimulus evaluation, discrimination, categorization, memory, and decision-making capacity [54]. Finally, the high sensitivity of SSR to hypoxia may be associated to the involvement of highly sensitive central areas in SSR electrogenesis [55], as well as to the high repetition rate of the stimuli. The fast emergence of SSR latency changes may suggest an impairment of central acoustic pathways as the main cause of P1 latency increase [2] at least in the presence of short exposure times and high speed of ascent.

## 5. Conclusion, pitfalls, and future perspectives

The available evidence on the effects of HA on the inner ear, collected in this scoping review, suggests that conditions of reduced oxygen (i.e., hypoxia) and atmospheric pressure (i.e., hypobaria) may exert detrimental effects on auditory function, such as elevation of hearing thresholds, lengthening of AER latencies, and reduction of evoked OAE. These alterations may be the results of the concurrent actions of multiple physiological and pathophysiological responses and adaptation mechanisms, which may act at different stages of the auditory pathway. Despite these indications, the actual magnitude, nature, and clinical significance (i.e., transient/reversible versus persistent nature of auditory impairment) of the induced changes remain not fully clarified, as well as the relationship between the observed changes, the acclimatization process and/or the development of altitude illness. Heterogeneity of the studies, in terms of differences in protocols (mainly exposure duration and magnitude, and absence/presence of hypobaria), effects of confounding physical factors (e.g., air pressure reduction effects on sound transmission) and diverse corrective countermeasures adopted, and the limited sample size of the studies are relevant limitations, which need to be addressed in future studies. The intrinsic stimulus and time-dependence of hypoxia response requires to be addressed by devising standardized protocols with defined spatio-temporal patterns of exposure to thoroughly characterize hypoxia effects on auditory function at different altitude levels and exposure duration. Differences in hearing function response to HH and NH beyond the physical effects on sound transmission need to be characterized. Attention could be focused on the relationship between alterations in cerebral blood flow autoregulation and auditory function, potentially by combing central circulation and auditory testing measures. Finally, although hypoxia may potentially act at different levels along the auditory pathway, the evidence available from this review does not allow to distinguish the contribution of peripheral and central mechanisms and their interaction. A multiparametric evaluation of auditory function through the combination of audiology and neuro-otology techniques may help to shed light on mechanisms acting at different stages and their interaction. While PTA may provide a simple and overall evaluation of auditory function, OAE analysis may be suitable to specifically detect cochlear alterations, while AER may track the downstream effects of these alterations in superior auditory pathways and their potential interaction with central mechanisms. In this view, simulated altitude settings may provide practical solutions for performing systematic and multiparameter evaluations, offering control and tuning of HA patterns and less restrictions on auditory assessment modalities. The progress in technological development favoring miniaturization and portability of the instruments, as well as the increased accessibility to HA areas, may further stimulate in-field research to corroborate results from simulated settings.

## Supporting information

S1 Checklist Preferred Reporting Items for Systematic reviews and Meta-Analyses extension for Scoping Reviews (PRISMA-ScR) checklist. (DOCX)
